# Reduction in gap junction intercellular communication promotes glioma migration

**DOI:** 10.18632/oncotarget.3407

**Published:** 2015-03-19

**Authors:** Qurratulain Aftab, Wun-Chey Sin, Christian C. Naus

**Affiliations:** ^1^ Department of Cellular & Physiological Sciences, Life Sciences Institute, The University of British Columbia, Vancouver, BC

**Keywords:** connexin, gap junction, glioma, migration

## Abstract

Glioblastoma Multiforme (GBM), an aggressive form of adult brain tumor, is difficult to treat due to its invasive nature. One of the molecular changes observed in GBM is a decrease in the expression of the gap junction protein Connexin43 (Cx43); however, how a reduction in Cx43 expression contributes to glioma malignancy is still unclear. In this study we examine whether a decrease in Cx43 protein expression has a role in enhanced cell migration, a key feature associated with increased tumorigenicity. We used a 3D spheroid migration model that mimics the *in vivo* architecture of tumor cells to quantify migration changes. We found that down-regulation of Cx43 expression in the U118 human glioma cell line increased migration by reducing cell-ECM adhesion, and changed the migration pattern from collective to single cell. In addition gap junction intercellular communication (GJIC) played a more prominent role in mediating migration than the cytoplasmic interactions of the C-terminal tail. Live imaging revealed that reducing Cx43 expression enhanced relative migration by increasing the cell speed and affecting the direction of migration. Taken together our findings reveal an unexplored role of GJIC in facilitating collective migration.

## INTRODUCTION

Glioblastoma multiforme (GBM) is a fatal glioma thought to arise from glial precursor cells or dedifferentiated astrocytes [1]. The World Health Organization (WHO) categorizes gliomas into four grades based on histological differences: grade 1: pilocytic astrocytoma; grade 2: diffuse astrocytoma; grade 3: anaplastic astrocytoma; grade 4: glioblastoma multiforme [2, 3]. The current treatment for glioma is resection of the tumor, followed by chemotherapy and radiation therapy [4, 5]. Even with such radical treatment patients with GBM suffer from recurring tumors which arise due to the invasive nature of glioma cells. In addition to the histological changes several molecular changes take place in the process of gliomagenesis [6–8].

Previous studies have shown a decrease in the expression of gap junction protein Connexin43 (Cx43) in high grade gliomas [9–12]. Our lab has also shown a decrease in Cx43 expression in high grade human gliomas in tumor microarrays [13]. Cx43 is the major gap junction protein in astrocytes; gap junctions directly link the cytoplasm of adjacent cells thus establishing a glial syncytium. Gap junctions between cells allow for passage of ions and small molecules such as Ca^2+^, cAMP, ATP, glucose and glutamate [14, 15]. The channel function of Cx43 has been shown to be regulated by phosphorylation of the C-terminal tail [16], which has several phosphorylation sites that serve as substrate to a number of kinases including Src, MAPK and PKC kinases [17].

Deciphering the role of Cx43 in glioma migration is complicated by the fact that two types of gap junctions exist *in vivo*; homocellular gap junctions formed between glioma cells, and heterocellular gap junctions formed between glioma and host cells. *In vivo* studies with rats have shown that glioma cells can establish gap junctional intercellular communication (GJIC) with astrocytes in the brain, which aids in their invasion [18, 19]. *In vitro* studies have shown that blocking the channel activity by carbenoxolone in GL15 human glioma cell line increased migration on extracellular matrix (ECM) proteins but decreased migration on astrocytes and brain slice cultures [20]. In addition, a reduction in Cx43 level in U251 human glioma cells usually showed an increase in migration, except when brain slices were used as a substrate [21, 22]. These findings show paradoxical roles for homocellular and heterocellular gap junctions.

In addition to the channel function of Cx43, the C-terminal tail has also been implicated in modulating migration. The C-terminal tail of Cx43 has several phosphorylation sites that are involved in regulating the protein's life cycle, channel function and interaction with the actin cytoskeleton [23–25]. We have previously shown that in rat C6 glioma cells the C-terminal tail was responsible for modulating migration [26]. In addition, the C-terminal tail has also been shown to cause changes in the actin cytoskeleton [27]. We have also shown that the C-terminal tail is needed for neuronal migration *in vivo* [28].

To understand the role of homocellular gap junctions in glioma migration we used short hairpin RNA to reduce endogenous Cx43 in the human glioma cell line U118. We show that reducing Cx43 increases migration, and also changes the migration pattern from collective to single cells. We used specific mutants to determine the domain of Cx43 responsible for influencing migration. The T154A is a dominant negative channel mutant that significantly blocks gap junction communication [29]. The C-terminal mutant TrCx43 truncates the tail at amino acid 242, eliminating the key phosphorylation sites and protein-protein interaction sites [27]. We found that obstructing the channel function increased migration. Our results highlight a new role for Cx43 in collective migration of glioma cells.

## RESULTS

### Reducing Cx43 changes the migration pattern from collective to single cell

**Figure 1:**
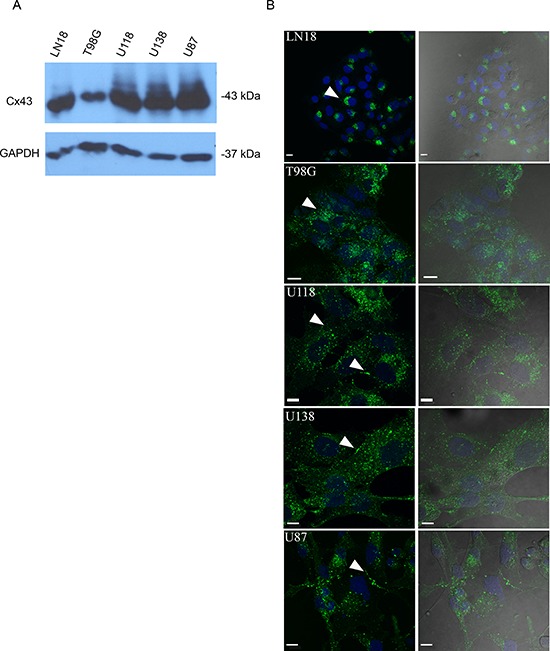
Cx43 expression and GJIC in various human GBM cell lines **(A)** Human GBM cell lines express Cx43 protein at varying levels as shown by Western blot. **(B)** LN18 and T98G show perinuclear localization of Cx43 (arrow). U118, U138, and U87 show Cx43 localizing to cell-cell contacts and in intracellular vesicles (arrows); scale bar = 10 μm. Anti-Cx43 (Sigma) antibody that targets the C-terminal tail of Cx43 was used to detect Cx43 protein.

**Figure 2:**
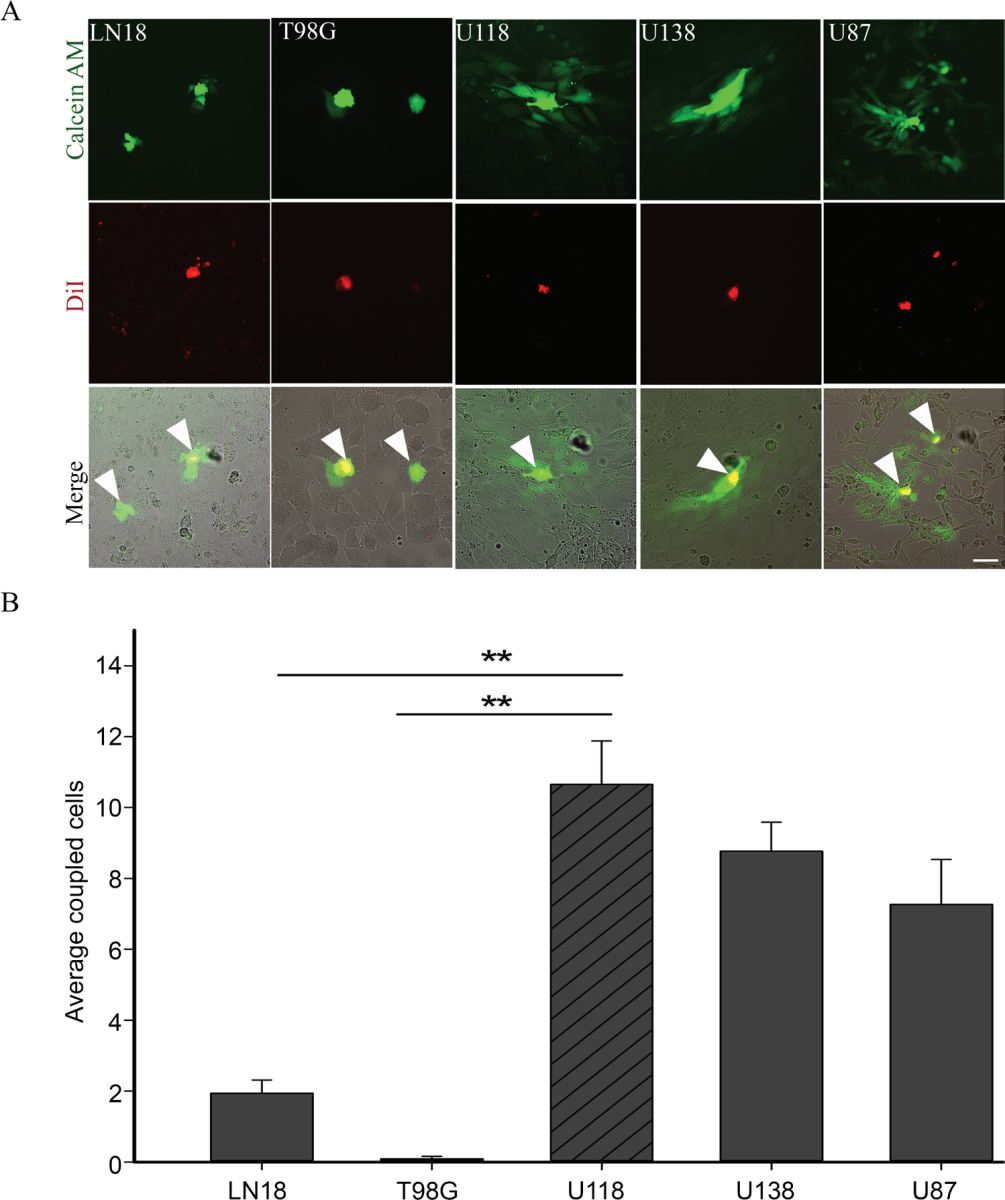
GJIC in human glioma cell lines **(A)** Preloading assay was used to examine GJIC in human glioma cell lines LN18, T98G, U118, U138 and U87. Donor cells (yellow because they have both dyes; see arrows) were labeled with DiI (red) and Calcein AM (green). Passage of Calcein to recipient cells through gap junctions labelled them green. **(B)** U118 cell line showed the highest level of GJIC as demonstrated by the passage of Calcein (green); scale bar = 50 μm. This experiment was repeated 3 times; **p < 0.001 determined by One Way Anova method followed by Dunn's method for multiple comparisons.

**Figure 3:**
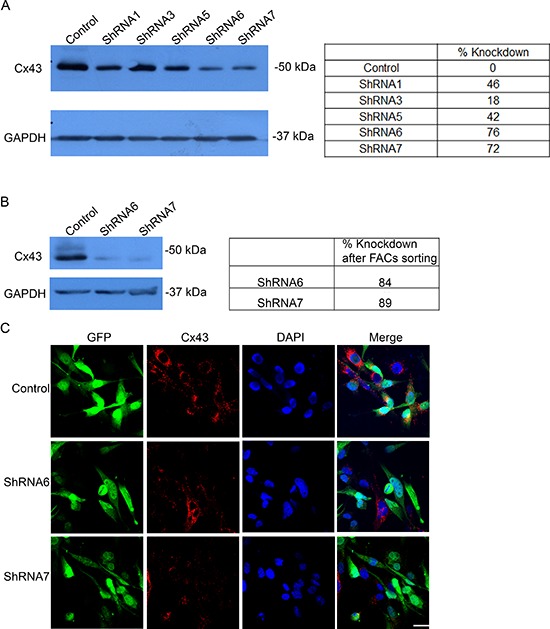
Expression of a panel of anti-Cx43 ShRNA constructs reduces Cx43 expression **(A)** Five different anti-Cx43 shRNA constructs were expressed in U118 cell line that produced varying levels of protein knockdown. ShRNA6 and ShRNA7 had the highest level of Cx43 protein reduction. **(B)** U118 cells expressing ShRNA6 and ShRNA7 constructs were FACS by GFP expression to obtain a higher population of cells expressing the ShRNA constructs shown by Western blot. ImageJ was used to quantify the intensity of the bands. The numbers represent the average of 3 blots. **(C)** Reduction in Cx43 was also observed by immunofluorescence using anti-Cx43 (Sigma) antibody; scale bar = 50 μm.

**Figure 4:**
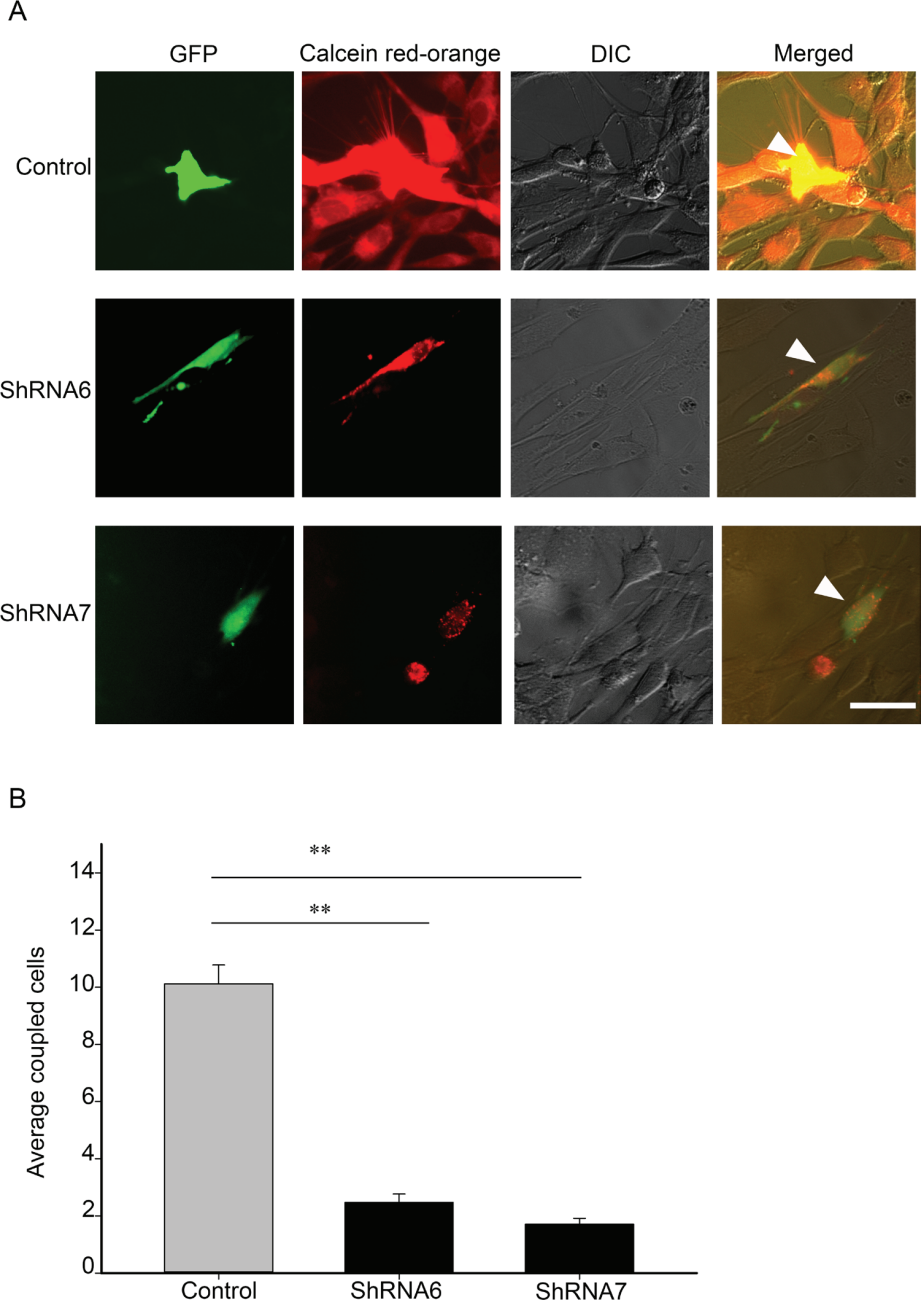
Reducing Cx43 expression decreases GJIC **(A)** Control, ShRNA6 and ShRNA7 cell lines (expressing GFP) were loaded with Calcein red-orange and appear yellow (arrow). Passage of Calcein red-orange to recipient parental U118 cells was an indication of GJIC; scale bar = 50 μm. **(B)** A significant reduction in coupled cells was quantified for both ShRNA6 and ShRNA7 cells indicating that GJIC had been reduced. This experiment was repeated 3 times; **p < 0.001 determined by One Way Anova method followed by Dunn's Method.

We screened a panel of human glioma cell lines with several of the key mutations found in GBM for Cx43 expression, subcellular distribution and GJIC (Figures 1 and 2). We observed varying levels of Cx43 protein expression in the glioma cell lines (Figure 1A). In most human glioma cell lines we examined, Cx43 localized at cell-cell contacts and in intracellular vesicles (Figure 1B). Cell lines that expressed higher levels of Cx43 also exhibited higher GJIC (Figure 2). The U118 cell line expressed Cx43 at cell-cell contacts and had the highest levels of GJIC (Figures 2A and 2B), indicating that it could form functional gap junctions. In addition, the U118 cell line has mutations in the p53 and PTEN genes, which are known to be important of gliomagenesis [30]. The aforementioned characteristics of the U118 cell line made it an excellent system to study Cx43 in glioma migration by performing loss of function and rescue experiments. We used wound healing and spheroid migration assays to investigate changes in migration due to Cx43 expression. The spheroid migration assay was carried out on fibronectin, an ECM protein that is upregulated in GBM facilitating invasion [31–35]. A panel of five ShRNA constructs that targeted different sites of the Cx43 gene were used to knockdown Cx43 expression in U118 human glioma cells (Figure 3A). Two different ShRNA constructs, ShRNA6 and ShRNA7, produced the highest degree of Cx43 protein expression knockdown in U118 cells as demonstrated by Western blot and immunocytochemistry (Figure 3B and 3C). Furthermore GJIC was significantly reduced in U118 cells expressing ShRNA6 and ShRNA7 constructs, and therefore were chosen for the migration studies (Figure 4A and 4B).

**Figure 5:**
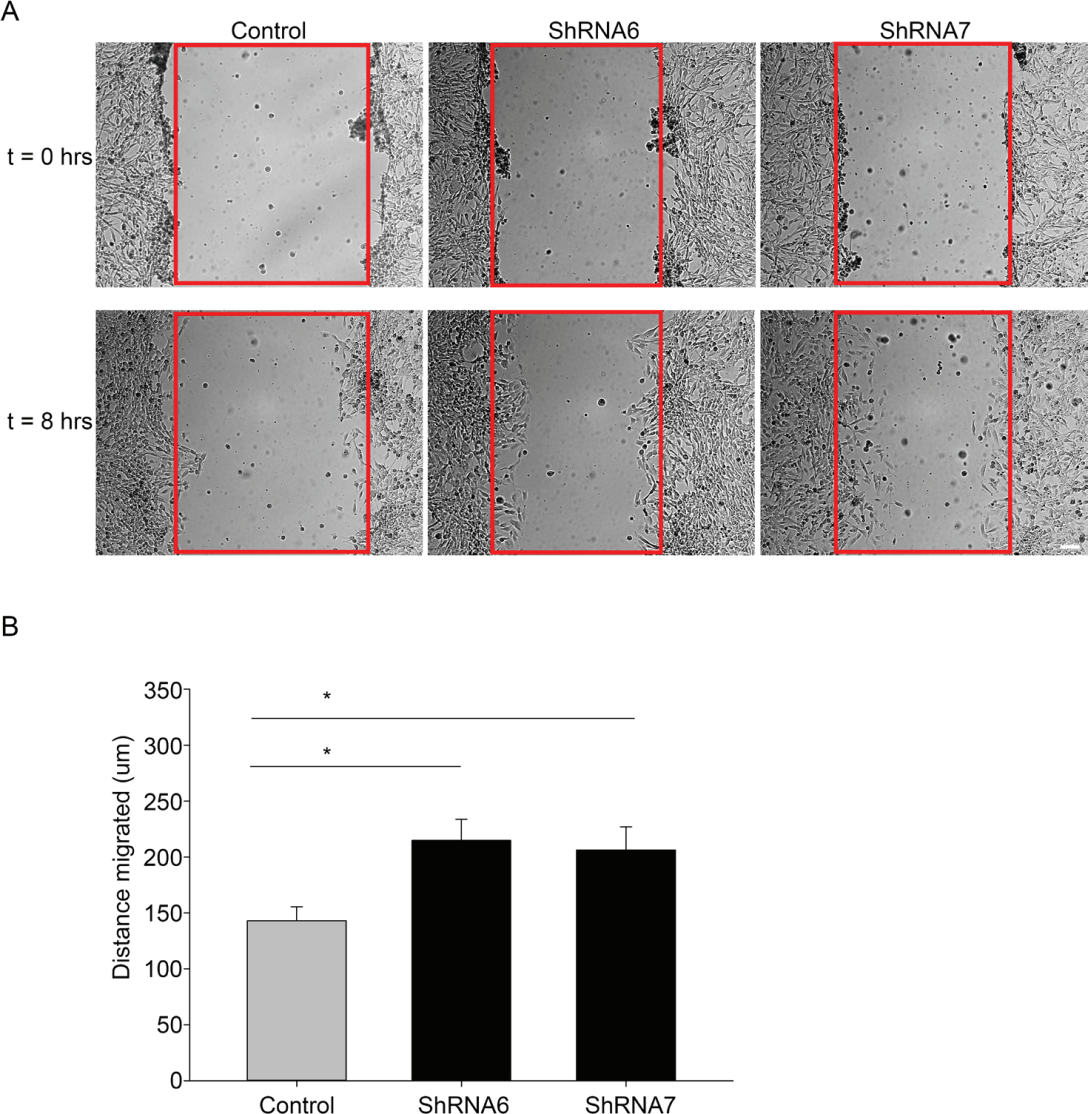
Reducing Cx43 expression increases migration in a wound healing assay **(A)** Control and shRNA 6 and 7 cells were grown to confluence and then scratched. The cells were imaged at t = 0 (when the cells were scratched) and at *t* = 8 hours. After 8 hours a higher number of ShRNA6 and ShRNA7 cells occupied the wound (wound highlighted in red). **(B)** Compared to control cells ShRNA6 and ShRNA7 increased migration by 50% and 45%, respectively. This experiment was repeated three times with *n* = 12 per condition; *p < 0.05 determined by One Way Anova method followed by Student-Newman-Keuls Method.

**Figure 6:**
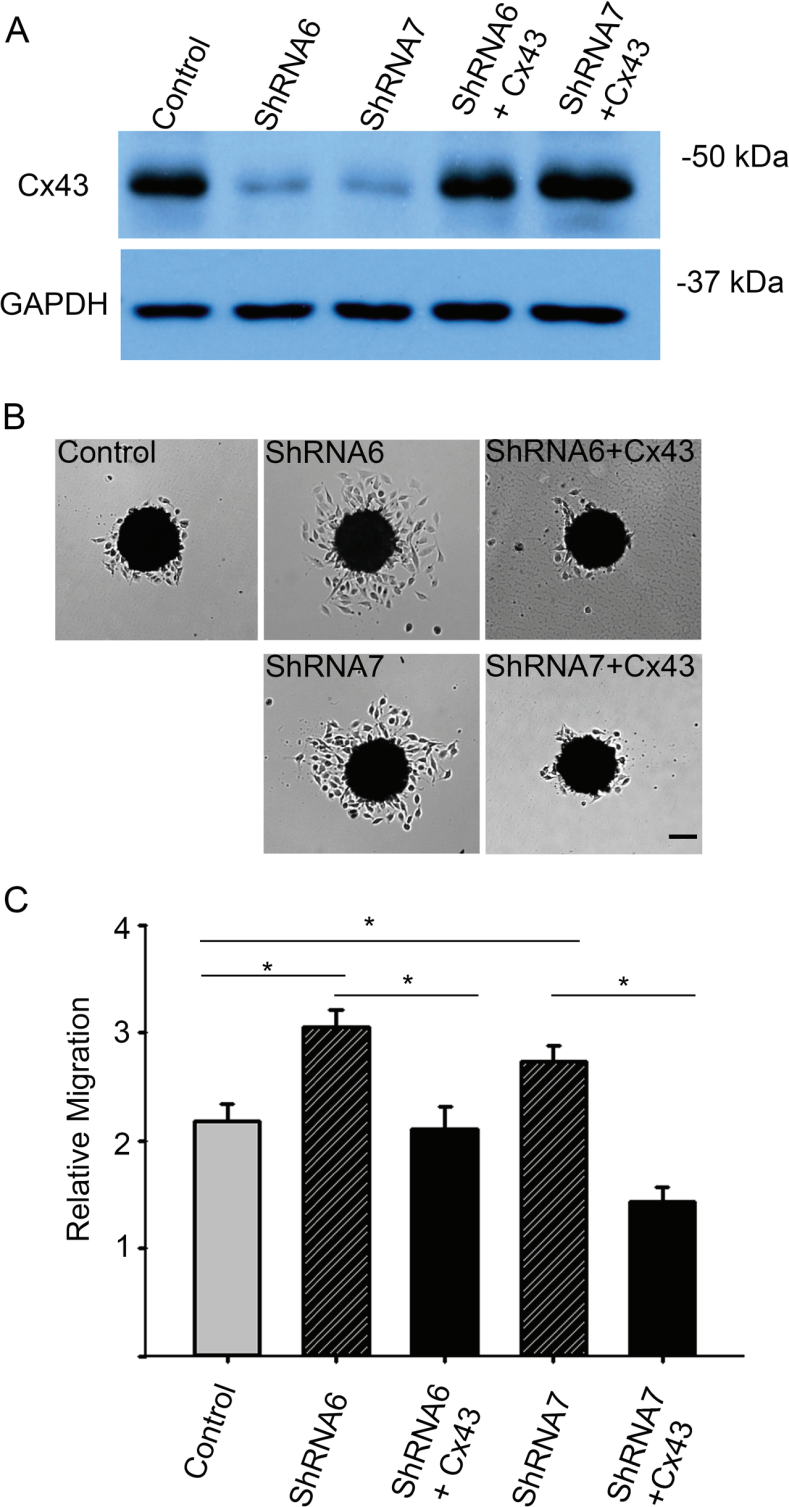
Reducing Cx43 expression increases migration in a spheroid migration assay **(A)** Western blot analysis shows successful expression of exogenous full-length Cx43 cDNA lacking the 3′UTR in U118-ShRNA6 and U118-ShRNA7 cells. Using densitometry we found the Cx43 expression in control and rescue cells to be 4 times higher than ShRNA6 and ShRNA7 cells (average of 3 Western blots). **(B)** Expression of Cx43 in the knockdown cells lowered migration level comparable to control cells. Scale bar = 100 μm. **(C)** Compared to control cells ShRNA6 and ShRNA7 cells increased migration 46% and 30%, respectively. The experiments were repeated three times with control (*n* = 81spheroids), ShRNA 6 (*n* = 81 spheroids), ShRNA 7 (*n* = 88 spheroids), ShRNA 6-Cx43 (*n* = 54 spheroids) and ShRNA 7-Cx43 (*n* = 54 spheroids). *p < 0.05 determined by One Way Anova method followed by Dunn's Method.

In the wound healing assay, U118 cells expressing ShRNA6 and ShRNA7 migrated faster than the control cells after 8 hours (Figures 5A and 5B). This result was confirmed with a spheroid migration assay that had the advantage of providing a 3D architecture similar to *in vivo* tumors with a core and a defined border [36]. In this assay, cells from ShRNA6 and ShRNA7 glioma spheroids migrated faster than control cells on fibronectin (Figures 6B and 6C). To confirm the increase in migration was due to the decrease in Cx43, we expressed full-length Cx43 in ShRNA6 and ShRNA7 cells (Figure 6A) and observed a reduction in the level of migration comparable to control cells (Figures 6B and 6C). Exogenous Cx43 proteins localized in intracellular vesicles and at cell-cell contacts (Supplementary Figure 1A), forming functional gap junctions (Figure 10). The ShRNA constructs were designed to bind to the 3′UTR region of the endogenous mRNA so they only target the endogenous Cx43 but have no effect on exogenous wild-type Cx43 expressed from the cDNA plasmid.

**Figure 7:**
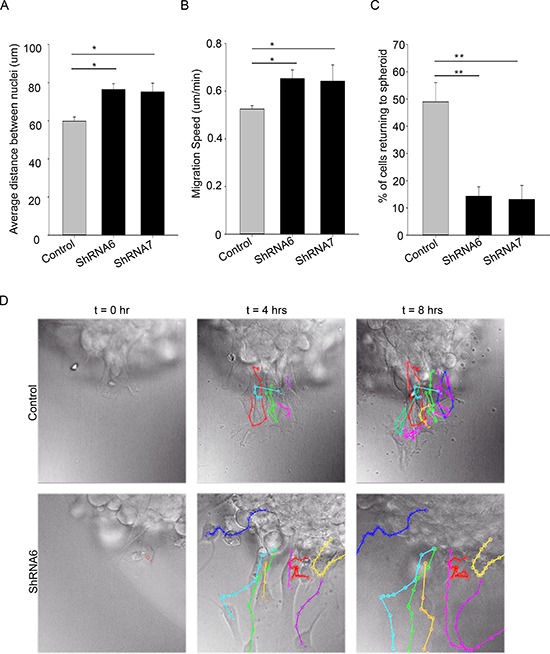
Reducing Cx43 expression changes cell migration pattern in U118 cells **(A)** Individual cells migrating away from the spheroid were tracked. Single cell tracking was done using ImageJ MtrackJ plugin; the nuclei of the cells were tracked. The distance between the nuclei of migrating cells was measured over an 8 hour time period. On average control cells migrated with shorter distance between them than ShRNA6 and ShRNA7 cells. **(B)** The speed of cells was calculated by measuring the total distance travelled by cells divided by the total time. ShRNA6 and ShRNA7 cells migrated faster than the control cells. **(C)** Reducing Cx43 influenced the direction of migration with a higher percentage of control cells returning to the spheroid (control = 49%, ShRNA6 = 14%, and ShRNA7 = 13%). **(D)** Cell tracks for control, ShRNA6 and ShRNA7 cells over 8 hours show difference in migration pattern. Control cells are migrating in a collective manner whereas the ShRNA and ShRNA7 cells are migrating in a more detached manner. The data shown here is from 3 experiments with Control *n* = 153 cells, ShRNA6 *n* = 174 cells, and ShRNA7 *n* = 195 cells; One way Anova (Sigma plot) followed by Holm-Sidak method to do pairwise multiple comparisons was used to calculate significance for distance between cells as they migrate, and the directionality; *p < 0.05, **p < 0.005 was considered significance. The student *t*-test was used to calculate the significance for cell speed; *p < 0.05 was considered significance. Scale bar = 50 μm.

Next, we examined whether there were changes in migration patterns between ShRNA6, ShRNA7 and control cells. Time lapse imaging of the spheroids revealed that the control cells differ in speed, directionality and cell-cell association when compared to ShRNA6 and ShRNA7 cells. The distance between adjacent nuclei was significantly shorter in the control cells compared to ShRNA6 and ShRNA7 cells, indicating a change in migration pattern from collective to single cell (Figures 7A and 7D). As expected, control cells migrated at a slower speed than ShRNA6 and ShRNA7 cells (Figure 7B). Interestingly, we observed a higher percentage of migrating control cells returning to the spheroid when compared to ShRNA6 and ShRNA7 cells (Figure 7C). We observed that after 4 and 8 hours the control cells were clustered close together and still near the spheroid edge whereas the knockdown cells were dispersed and had left the field of view (Figure 7D, Supplementary Movies 1 and 2).

### Reduction in gap junctional intercellular communication increases migration

**Figure 8:**
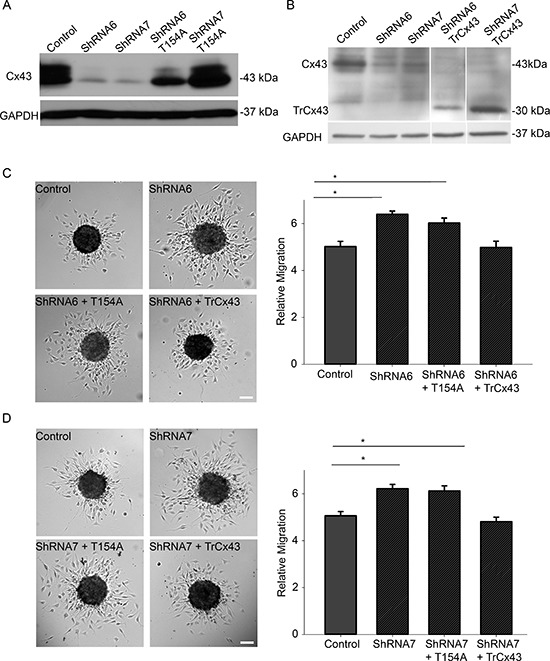
A reduction in Cx43 channel activity increases migration Western blot analysis was done to examine the successful expression of mutants T154A and TrCx43 in ShRNA6 and ShRNA7 cells. **(A)** We used anti-Cx43 (Sigma) that targets the C-terminal domain of Cx43 to detect mutant protein T154A. Using densitometry (ImageJ) we found Cx43 expression in control cells to be 16 times higher than ShRNA6 and ShRNA7 cells. Cx43 expression in ShRNA6-T154A and ShRNA7-T154A cells was 9 and 16 times higher, respectively. **(B)** Since the TrCx43 mutant lacks the C-terminal tail we could not use the Sigma anti-Cx43 antibody we used for detection of wild-type Cx43 or T154A. We used P1E11 anti-Cx43 antibody (Fred Hutchinson Cancer Research Center) that targets the N-terminal of Cx43 to detect TrCx43 expression in ShRNA6 and ShRNA7 cells. Since the TrCx43 is lacking the C-terminal tail it is of a smaller molecular weight, predicted to be 30 kDa. Cx43 expression in control cells was 6 times higher than ShRNA6 and ShRNA7 cells. Cx43 expression in ShRNA6-TrCx43 and ShRNA7-TrCx43 cells was 3 and 6 times higher, respectively. Please note that extraneous lanes were removed from the blot. The expression of T154A mutant in the ShRNA6 and shRNA7 cells did not reduce migration levels to that of control cells. An increase in migration comparable to the knockdown cells was observed **(C)** ShRNA6 (27%), ShRNA6-T154A (20%), **(D)** ShRNA7 (23%) and ShRNA7-T154A (21%). The TrCx43 mutant produced migration levels similar to control cells. The experiment was repeated three times with Control (*n* = 61spheroids), ShRNA6 (*n* = 55 spheroids), ShRNA6-T154A (*n* = 58 spheroids), ShRNA6-TrCx43 (*n* = 61spheroids); Control (*n* = 62 spheroids), ShRNA7 (*n* = 61 spheroids), ShRNA7-T154A (*n* = 60 spheroids), and ShRNA7-TrCx43 (*n* = 58 spheroids). One way Anova followed by Dunn's Method to do pairwise multiple comparisons was used to calculate significance. *p < 0.05 was considered significant. Scale bar = 100 μm.

To determine the function of Cx43 responsible for mediating migration, we expressed the dominant negative channel dead mutant T154A in ShRNA6 and ShRNA7 cells (Figure 8A). We found that expressing the T154A mutant significantly reduced GJIC (Figure 10). Unlike the expression of full length wild-type Cx43, expression of the T154A mutant in ShRNA6 and ShRNA7 cells did not reduce migration levels to that of control cells. Rather we observed migration levels comparable to ShRNA6 and ShRNA7 cells (Figures 8C and 8D). This implies that for a cell to increase its migration rate it must either decrease Cx43 channel activity or reduce Cx43 protein expression; this coincides with the reduction in Cx43 protein expression observed in high grade glioma.

The C-terminal domain of Cx43 has been implicated in modulating cell migration and is known to interact with cytoskeletal proteins. To determine if the C-terminal domain played a role in glioma migration we used a mutant in which the C-terminal tail has been truncated after amino acid position 242 (TrCx43) (Figure 8B). TrCx43 proteins localized in intracellular vesicles and at cell-cell contacts (Supplementary Figure 1B), forming functional gap junctions (Figure 10). This mutant lacks the phosphorylation and many protein-protein interaction sites but still retains a significant level of channel activity [13, 27]. Indeed we also found the TrCx43 mutant to produce GJIC comparable to control cells (Figure 10). The expression of the TrCx43 mutant in ShRNA6 and ShRNA7 cells produced migration levels comparable to control and ShRNA6-Cx43 and ShRNA7-Cx43 (Figures 8C and 8D). Our findings suggest that the association of Cx43 C-terminal tail with the cytoskeleton is probably not critical in controlling migration in U118 glioma cells. It is interesting to note that the TrCx43 mutant made functional gap junction channels and rescued the knockdown phenotype as well as the wild-type, indicating that a significant reduction in channel activity is needed to increase cell migration.

**Figure 9:**
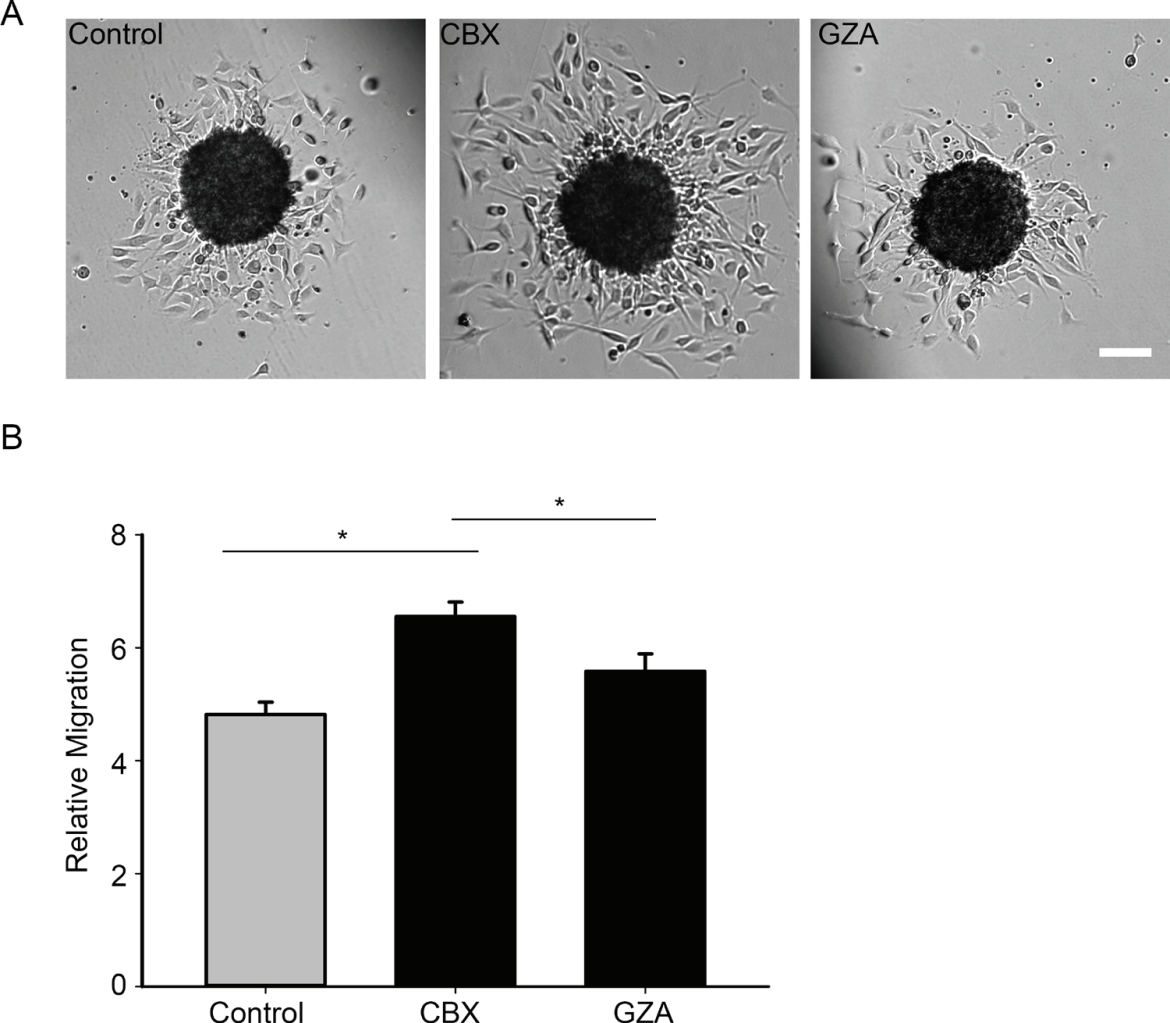
Blocking gap junction communication with a pharmacological inhibitor increases migration **(A)** Spheroid migration assay was carried out on control cells and cells that were treated with 150 μM of CBX, after 8 hours the cells were imaged and migration quantified **(B)** Inhibiting gap junction communication with CBX (150 μM) increases migration levels by 31%. The inactive analog GZA was unable to produce the same degree of increase in migration. This experiment has been repeated 3 times with *n* = 44 spheroids for each condition. *Student *t*-test was performed to calculate significance, *p < 0.05 was considered significance. Scale bar = 100 μm.

**Figure 10:**
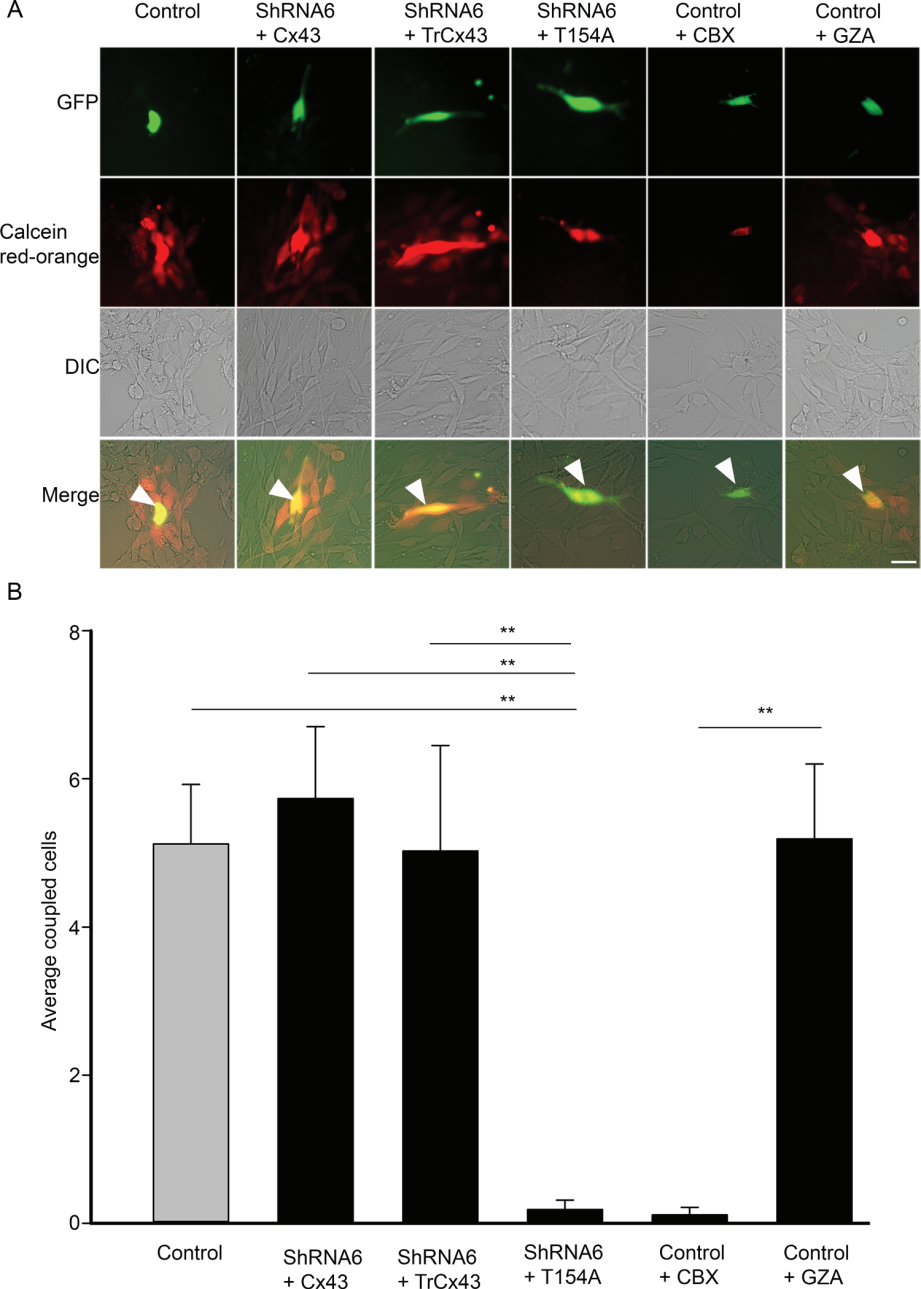
Cx43 mutant T154A reduces GJIC **(A)** Control, ShRNA6 cells expressing wild-type Cx43, TrCx43 and T154A mutants (expressing GFP) were loaded with Calcein red-orange and appear yellow (arrow). Passage of Calcein Red-Orange to recipient parental U118 cells was an indication of GJIC; scale bar = 50 μm. **(B)** A significant reduction in coupled cells was quantified for both ShRNA6+T154A and control cells treated with CBX indicating that GJIC had been reduced. This experiment was repeated 3 times; **p < 0.001 determined by One way Anova method followed by Dunn's Method.

To further confirm that the channel function is involved in migration, we used a complementary approach by carrying out the spheroid migration assay on parental cells in the presence of a gap junction blocker carbenoxolone (CBX) (150 μM) and its inactive analog glycyrrhizic acid (GZA) (150 μM). The presence of CBX increased migration by 31% when compared to untreated control cells (Figure 9A and 9B). CBX reduced GJIC, as did the T154A mutant, whereas treatment with GZA did not affect GJIC (Figure 10). Taken together, our results highlight that it is the gap junction channel function of Cx43 that is playing a role in cell migration.

### A reduction in Cx43 decreases cell-ECM adhesion

**Figure 11:**
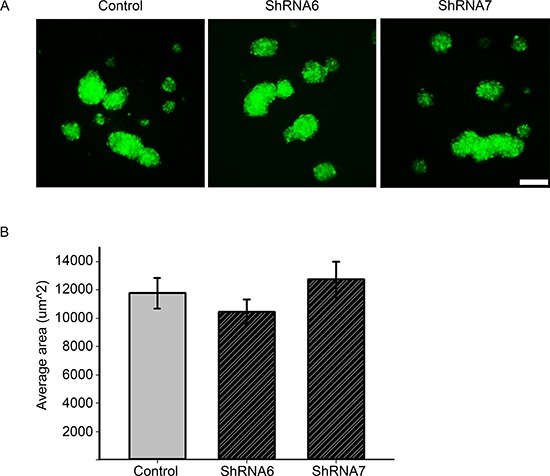
Intercellular adhesion is not affected after knocking down Cx43 **(A)** Control, ShRNA6, and ShRNA7 cells were seeded in agar coated 96 well plates for 24 hrs. GFP positive aggregates were imaged since that is a marker for ShRNA construct expression. Control cells produced aggregates of comparable size to ShRNA6 and ShRNA7 cells. **(B)** The average size of aggregates showed no significant difference. The size of the aggregates was quantified by ImageJ software. Only aggregates > 3000 μm^2^ were counted in the analysis. The experiment was repeated three times with *n* = 72, and 500 aggregates per condition. Scale bar = 100 μm.

We observed a switch from collective to single cell motility when Cx43 is reduced and hence it is possible that cell-cell adhesion is affected. Since the docking of Cx43 hemichannels is known to increase intercellular adhesion [19], we examined whether the increase in migration is due to a weakening of intercellular adhesion. To examine changes in intercellular adhesion we carried out a slow aggregation assay in which there is no substrate to which cells can adhere, thus only testing cell-cell adhesion. We found no significant change in the average size of the aggregates between control and ShRNA6 and ShRNA7 cells (Figures 11A and 11B). A reduction in Cx43 has been shown to change the subcellular localization and expression of the cell adhesion molecule N-cadherin [37], however we did not observe a change in the expression nor the subcellular localization of N-cadherin (Supplementary Figure 2B). Our results suggest that reducing Cx43 expression in glioma cells does not affect their ability to form intercellular adhesions; however it is still unclear whether the stability of the adhesions is affected.

**Figure 12:**
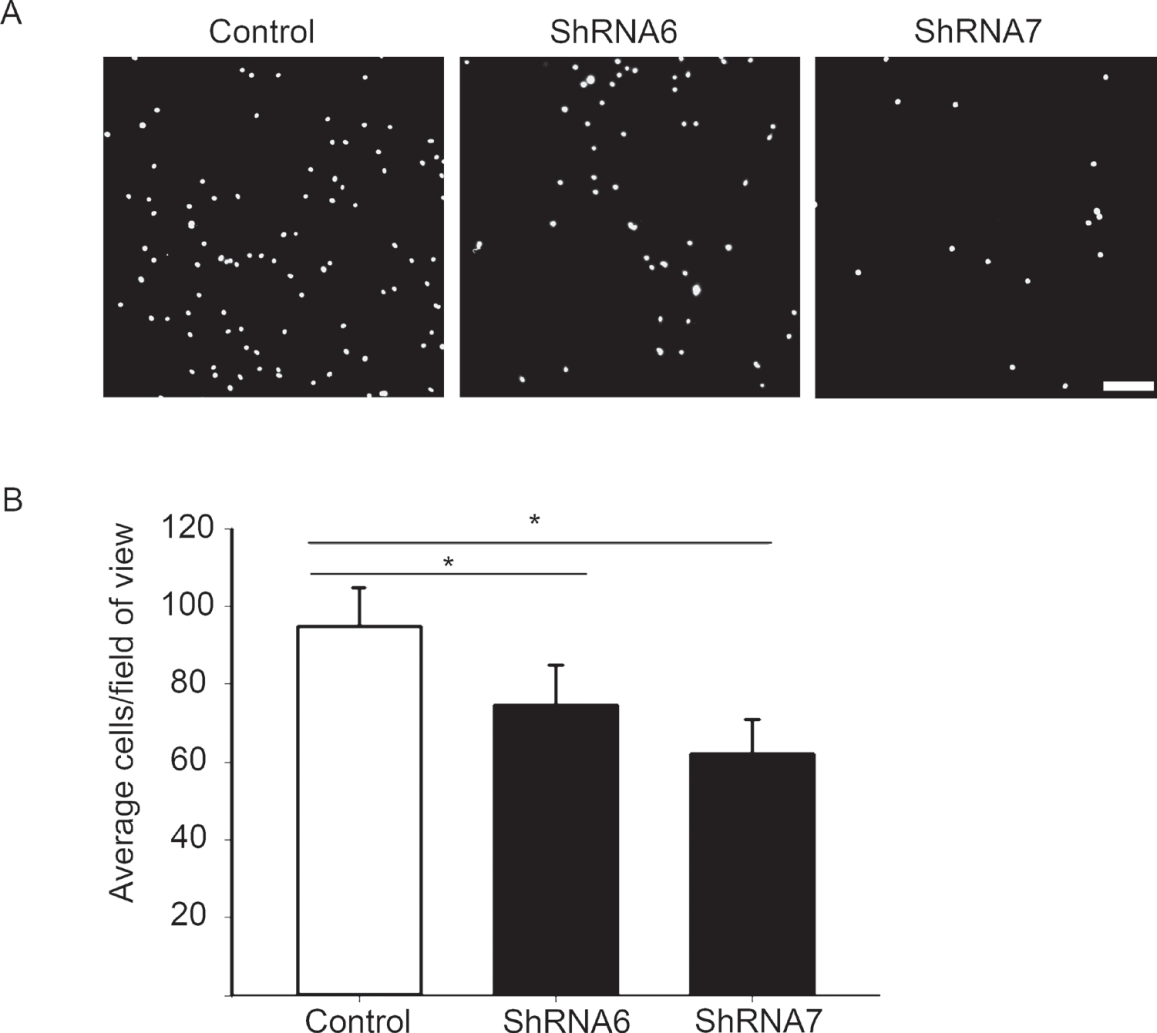
A reduction in Cx43 decreases cell adhesion to fibronectin **(A)** A high number of control cells adhered to fibronectin coated coverslips versus ShRNA6 and ShRNA7 cells (DAPI in white). **(B)** Adhesion to fibronectin was reduced in ShRNA6 and ShRNA7 cells by 21% and 34%, respectively. The experiment were repeated 4 times with *n* = 13 for all conditions. *p < 0.05 was determined by One Way Anova (Student-Newman-Keuls Method). Scale bar = 100 μm.

In addition to intercellular adhesions, the strength of cell-ECM adhesion also affects how fast a cell will migrate [38, 39]. Therefore we investigated whether decreasing Cx43 expression leads to changes in cell-ECM adhesion by carrying out an adhesion assay with fibronectin as the ECM component. We noticed a significant decrease in adhesion for ShRNA6 and ShRNA7 cells compared to control cells after 1 hour (Figures 12A and 12B). Western blot analysis showed no change in the expression of total β1-integrin, FAK and phospho-FAK, key proteins in cell-ECM adhesion (Supplementary Figure 2A). Immunocytochemistry analysis of active β1-integrin, FAK and phopsho-FAK on spheroids showed no change in the subcellular localization (Supplementary Figure 2B). Our findings suggest that decreasing Cx43 decreases cell adhesion to fibronectin which may be involved in the increase migration observed.

## DISCUSSION

Aggressive high grade gliomas have been reported to exhibit low expression of Cx43 [40]. A feature of aggressive glioma is their enhanced ability to migrate to healthy parts of the brain. The role of Cx43 in glioma migration is not clearly defined. There is a general agreement that heterocellular Cx43 gap junctions formed between glioma-astrocytes and glioma-endothelial cells facilitate invasion [18–20, 41]. However, the role of homocellular Cx43 gap junctions between glioma cells is debatable and dependent on the system used [20, 21, 26, 27]. Overall the role of homocellular gap junctions in glioma migration has been examined by using 2D monolayer migration assays. Therefore, we decided to use the stringent approach of loss-of-function and rescue experiments in a 3D spheroid model that better mimics invading glioma cells exiting from a hypoxia core [36]. To further create an assay more akin to *in vivo* we used the ECM protein fibronectin which is up-regulated in GBM as the substratum for the cells [31]. We reduced endogenous Cx43 expression by two independent ShRNAs and then rescued it by expressing wild-type or mutated/truncated Cx43; using two different migration assays we show that decreasing Cx43 expression increases glioma migration.

Our study is the first to show that reducing Cx43 in glioma cells changes the mode of migration from collective to single cell motility. In fact, the same phenotype has been observed in a study in breast epithelial cells, where reducing Cx43 expression increased cell migration by facilitating single cell motility rather than collective [42]. An increase in cell speed correlates with a switch from collective to single cell motility [43], and indeed reducing Cx43 expression increased the speed of glioma cells thus explaining the increase in relative migration. We also observed a higher percentage of control glioma cells returning to the spheroids which suggests that reducing Cx43 allows the cells to migrate in a persistent directional manner and cover a larger area.

Cx43 is a multi-modular protein that couples cells electrically and metabolically through its channel and physically through its extracellular loops. Given that previous studies have implicated Cx43 as an adhesion molecule it is possible that the expression of Cx43 at the cell-cell junctions could be aiding cell adhesion in collective migration [19, 44]. The protein's C-terminal tail has phosphorylation sites that regulate the life cycle of the protein and also affect the channel function [20, 25, 26]. As well, the C-terminal tail is known to interact with cytoskeletal proteins [24, 45] and influence neuronal and glioma migration [26, 28]. The C-terminal tail has been shown to influence actin cytoskeletal dynamics by its interaction with F-actin or with actin binding proteins such as drebrin and cortactin [46, 47]. In contrast, the mechanism by which Cx43 gap junction channels may affect the actin cytoskeleton is still unclear. The ability of the C-terminal truncated mutant (TrCx43) to produce migration levels similar to control cells suggest that the C-terminal is not mediating migration and thus the rearrangement of the actin cytoskeleton may not be the underlying mechanism for the observed migration changes. Indeed this is strengthened by our observation of no obvious changes in the actin cytoskeleton and cell morphology in control and knockdown cells (Supplementary Figure 3).

**Figure 13:**
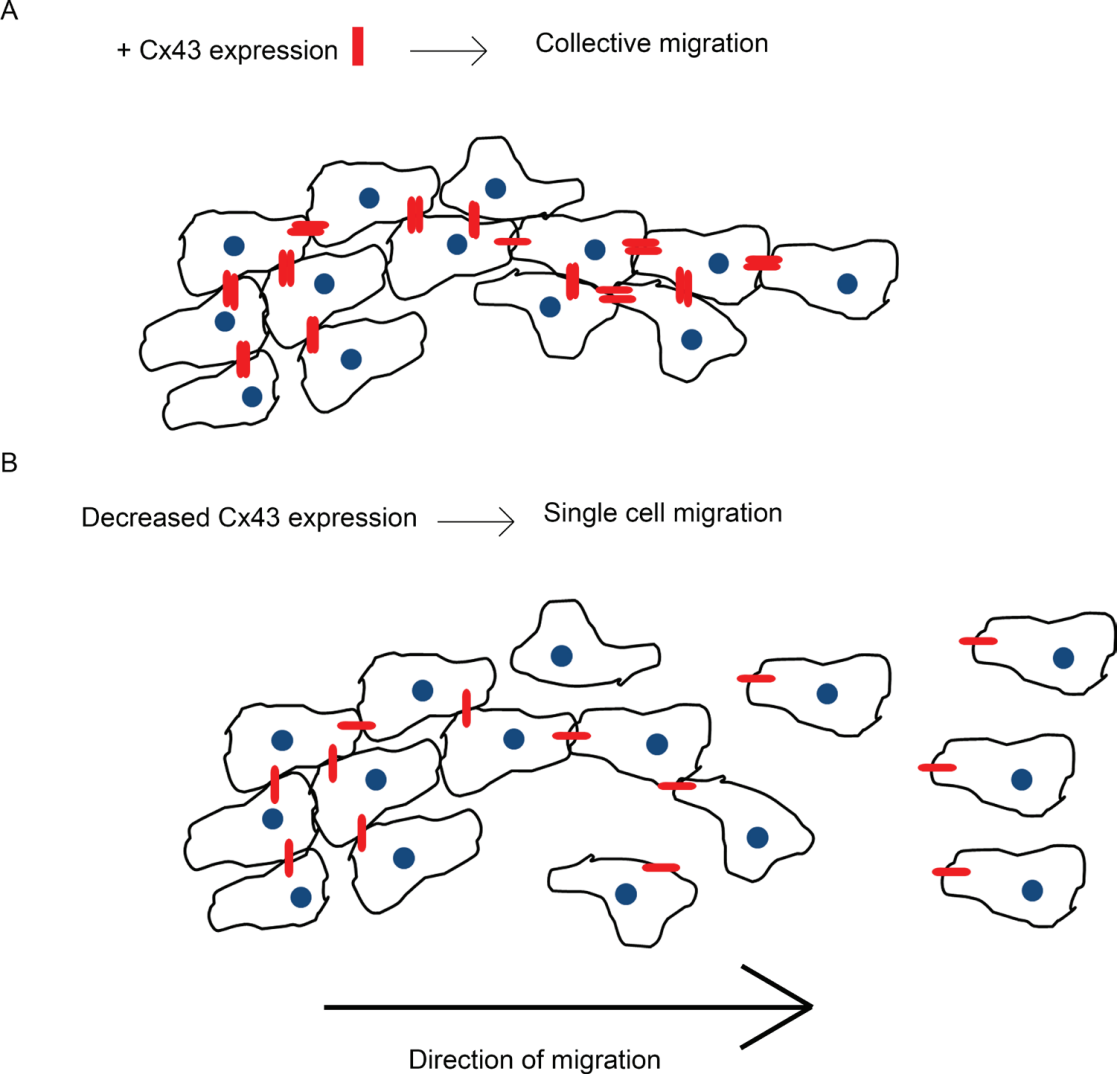
Cx43 expression facilitates collective migration This schematic is a summary of our findings from the *in vitro* 3D spheroid migration assay. **(A)** Endogenous expression of Cx43 protein forms gap junctions between glioma cells (Cx43 in red). Gap junctions physically connect the cells and allow exchange of molecules which facilitates collective migration. **(B)** Decreasing Cx43 expression in glioma cells weakens the connection between them and reduces the passage of molecules thus promoting single cell motility.

We showed that blocking the channel function with a specific mutant (T154A) and a chemical blocker (CBX) increased migration suggesting that a significant reduction in GJIC is required to increase migration. Gap junctions can pass small molecules that are important in many signalling pathways, such as glucose, IP_3_ and ATP, as well as ions such as Ca^2+^ and H^+^ [48, 49]. The passage of such important metabolites and second messengers through gap junctions assist cells in tissues to maintain homeostasis and to function in a coordinated manner. Interestingly, adrenal cells transfected with Cx43-GFP migrate in a collective sheet pattern and retain gap junctions as they migrate [50, 51]. Thus it stands to reason that the presence of gap junctions between glioma cells could be aiding to coordinate collective cell migration and when gap junctions are absent the cells transition to single cell motility (Figure 13).

A positive correlation between Cx43 expression and cell-cell adhesion in the rat glioma C6 cell line has been reported previously [19], however when we examined intercellular adhesion in the various U118 cells using an aggregation assay we observed no difference. Our method measured the ability of cells to form adhesions but did not measure the strength of the intercellular adhesions. Hence it is possible that reducing Cx43 weakens intercellular adhesions, permitting the cells to detach and move as single cells. Although we did not observe changes in the subcellular localization or expression of N-cadherin, the main protein that facilitates intercellular adhesion, it remains a possibility that Cx43 affects collective migration by influencing the dynamics of intercellular adhesion molecules.

Cell-ECM adhesions are formed when integrins on the cell surface bind ECM proteins such as fibronectin; they facilitate migration by providing traction and organizing signalling pathways that are involved in migration. A decrease in cell-ECM has been shown when Cx43 is decreased in the U251 human glioma cell line [21]. Similarly, we found that glioma cells with reduced Cx43 were less adhesive to fibronectin than control cells. Fibronectin specifically bind to α5β1 integrin receptors and expression of this heterodimer has been associated with poor prognosis and tumor aggressiveness [52]. Expression and turnover of integrins influence the strength of cell-ECM adhesions [53]; however we found no change in the expression of total β-1 integrins (Supplemental Figure 2A). We also did not observe changes in the localization of active β-1 integrin (Supplemental Figure 2B); hence it is possible that there are changes in β-1 integrin turnover that could be promoting increased migration. Cell-ECM adhesion provides traction for the cell and affects the speed of migration [38, 53]; our results suggest that reducing Cx43 lessens the traction on fibronectin allowing cells to migrate faster.

Our study highlights a new role for Cx43 as a determinant of migration patterns in gliomas. In addition we show that this is mediated by the gap junction channel function of Cx43. Collective migration requires cells to remain physically connected and chemically synchronized; given that gap junctions meet both of these criteria suggests that Cx43 may be an important protein in this mode of migration.

## MATERIALS AND METHODS

### Cell lines and shRNA constructs

Human glioma U118 cell line was obtained from American Type Culture Collection (ATCC). GIPZ lentiviral Control and shRNA constructs 1, 3, 5, 6, 7 were obtained from Open Biosystems. All constructs targeted different parts of the 3′UTR. The mature anti-sense sequence for construct 1 was TCAGTAATAGCATTACTGC, construct 3 was AATGTAAACACCATATTGG, construct 5 was TAAGGACAATCCTCTGTCT, the mature anti-sense sequence for construct 6 was TGAGTACCACCTCCACCGG, and construct 7 was TAAATACCAACATGCACCT. The shRNA constructs have the GFP gene for a selection tool. Fugene transfection reagent was used to deliver the shRNA constructs to the cells. The transfected cells were selected by addition of puromycin (0.5 μg/ml) and the expression of GFP. The cells were sorted on a BD FACS AriaIIu machine (UBC Flow Cytometry Facility) using an 85um nozzle at 25psi. The cells were identified based on their forward and side scatter (both physical parameters) and GFP was detected using a 530/30 nm filter against an empty channel, PerCP-Cy5.5 (695/40 nm), to detect potential auto-fluorescence.

### Site directed mutagenesis and expression of wild-type and mutant Cx43

Cx43, Cx43-TrCx43 and Cx43-T154A cDNAs were inserted into pMSCVpuro vectors (CLONTECH Laboratories). The point mutation for Cx43-T154A cDNA was generated by using the Qiagen site directed mutagenesis kit. The primer sequence for Cx43-T154A was GGCTTGCTGAGAGCCTACATCATCAGCATCC (mutation underlined). The mutant construct was validated by sequencing (NAPS Unit, Biotechnology Laboratory, University of British Columbia). Plasmids pMSCVpuro-Cx43, pMSCVpuro-Cx43-TrCx43, pMSCVpuro-Cx43-T154A, and the empty vector pMSCVpuro were transfected in to HEK293 packaging cell line using Lipofectamine 2000 (Invitrogen), and the viral titres were collected as per Crespin [27]. The viral titres were placed on U118 cells expressing ShRNA6 and ShRNA7. Puromycin (1.5 μg/ml) was used to select the cells expressing the constructs. Cells were used 1 week after successful transfection. TrCx43 mutant is truncated at amino acid position 242 [27].

### Western blot

Cells were cultured to confluence in 100 mm dishes. At confluence the cells were washed with cold PBS and lysed in 500 μL radioimmune precipitation lysis buffer (RIPA) containing phosphatase inhibitors (Sigma) and protease inhibitors (Roche) [26]. DNA was sheared by sonication. Protein was quantified by using the colorimetric BCA Protein Assay Kit (Pierce) to determine the protein concentration of the samples. 30 μg of protein was loaded on to 10% acrylamide gels. Gel electrophoresis was carried out at 100 V. The antibodies used were anti-Cx43 (rabbit, Sigma, 1:4000; mouse P1E11 clone, Fred Hutchinson Cancer Research Center, 1:50), and anti-GAPDH (mouse, HyTest Ltd., 1:10, 000). The anti-Cx43 antibody from Sigma binds the C-terminal tail. The anti-Cx43 antibody P1E11 binds the N-terminal of Cx43 at residues 1–20. Densitometry to quantify changes in protein expression was performed using the ImageJ software (NIH).

### Immunocytochemistry on monolayer

Cells on coverslips were fixed in 4% paraformaldehyde in PBS buffer for 10 minutes at room temperature. The cells were rinsed with PBS twice and incubated in blocking solution (2% BSA + 0.3% Triton X-100) for 30 minutes. Samples were incubated with primary antibodies in working solution (1% BSA + 0.3% Triton X-100) for 1 hour at room temperature. The antibodies used were anti-Cx43 (rabbit, Sigma, 1:400; mouse P1E11 clone, Fred Hutchinson Cancer Research Center, 1:50). Coverslips were washed three times (10 minutes each) with PBS. Coverslips were then incubated with appropriate secondary antibodies, after which they were washed and mounted on microscope slides with Prolong Gold containing DAPI. Confocal microscopy was used to collect images of the cells.

### Spheroid migration assay

Cells were cultured as spheroids in low attachment round bottom 96 well plates. Spheroids were grown in DMEM-F12 with B27, EGF, FGF, glutamate for 2 days. The spheroids were then seeded on fibronectin (10 μg/ml) coated coverslips in the defined media mentioned above and allowed to migrate for 8 hours. After 8 hours the spheroids were imaged and the migration was quantified by subtracting the area of spheroid from area of migrated cells and dividing by area of spheroid. The method was modified from [54, 55].

### Wound healing migration assay

1 × 10^6^ cells were seeded in 6 well plates (uncoated, since scratch would perturb ECM coating) in serum free media; on day 2 the monolayer of cells was scratched. The scratch was imaged at t = 0 hours and at t = 8 hours. Distance migrated was calculated by subtracting the length migrated at t = 8 hours from the starting point at t = 0 hours. Methods modified from [26].

### Adhesion assay

Cell-ECM adhesion was examined by performing an adhesion assay using methods modified from [56, 57]. Cells were serum starved overnight and seeded the next day on fibronectin (10 μg/ml) coated coverslips in serum free media. After an hour the coverslips were washed and agitated at 300 rpm for 5 minutes twice and then fixed and stained with DAPI. Epifluorescence microscopy was used to image the adhered cells. Image J was used to count the adhered cells.

### Aggregation assay

Cells were serum starved overnight. Single cells were seeded on agar coated plates and incubated for 24 hours at 37°C in serum free media. Images of the aggregates were acquired through epifluorescence microscopy and the size of the aggregates was quantified by ImageJ software. Only aggregates > 3000 um^2^ were counted in the analysis. This method was modified from [58].

### Preloading assay on human glioma cell lines

LN229 control and mutant cell lines were grown until confluence. GJIC was assessed using the preloading assay [59]. Briefly, parental U118 cells were cultured to confluence to serve as recipient cells. Control, ShRNA6 and ShRNA7 cells were loaded with preloading solution (1 μl of Calcein AM and DiI to 1 ml of 0.3 M glucose), and incubated for 20 minutes at 37°C. The loading solution was removed and the cells were washed three times with 0.3 M glucose. Cells were trypsinized and titrated until they were completely detached. Donor cells were seeded on to the recipient cells and incubated for 2 hours at 37°C. Epifluorescence microscopy was used to image the coupled cells. The donor cells appeared yellow due to the presence of Calcein AM (green) and DiI (red). Passage of Calcein to the recipient cells labelled them green.

### Preloading assay on cells expressing shRNA and mutants

GJIC was assessed using the preloading assay [59]. Briefly, parental U118 cells were cultured to confluence to serve as recipient cells. Control, ShRNA6 and ShRNA7 cells were loaded with preloading solution (1 μl of Calcein Red-Orange to 1 ml of 0.3 M glucose), and incubated for 20 minutes at 37°C. The loading solution was removed and the cells were washed three times with 0.3 M glucose. Cells were trypsinized and titrated until they were completely detached. Donor cells were seeded on to the recipient cells and incubated for 2 hours at 37°C. Epifluorescence microscopy was used to image the coupled cells. The donor cells appeared yellow due to the expression of GFP and presence of Calcein Red-Orange. Passage of Calcein Red-Orange to the recipient cells labelled them red.

## SUPPLEMENTARY FIGURES AND MOVIES


