# Supplementary material for: Reduction in gap junction intercellular communication promotes glioma migration
Source: Oncotarget. 2015 Mar 19;6(13):11447–64. doi: 10.18632/oncotarget.3407 (PMC4484468; doi:10.18632/oncotarget.3407)
Supplement: Supplementary file 1 [file oncotarget-06-11447-s001.pdf]

SUPPLEMENTARY FIGURES

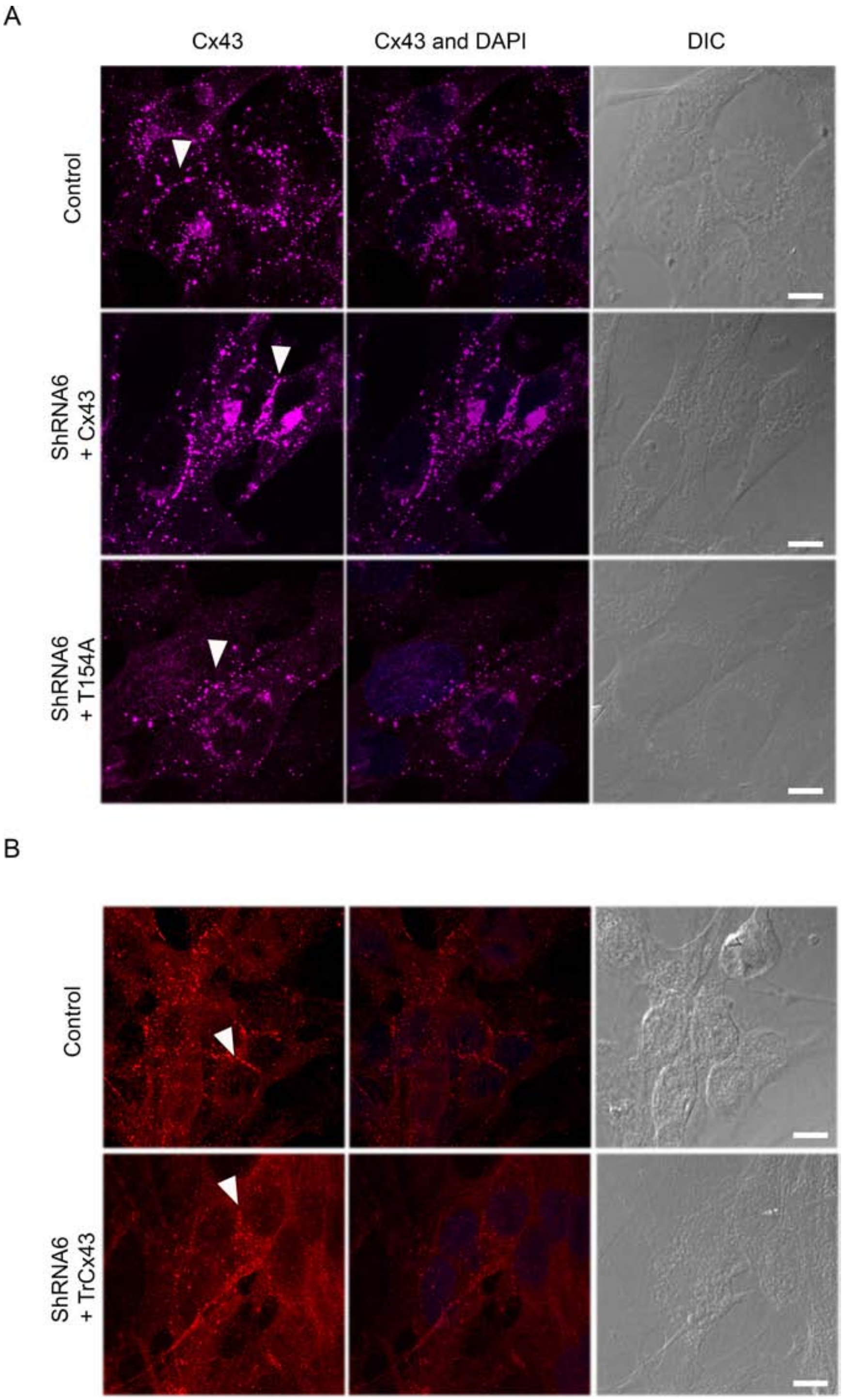

Supplementary Figure S1: Subcellular localization of Cx43 mutants in ShRNA6 cells. A. Immunofluorescence on control, ShRNA6+Cx43, and ShRNA6+T154A using anti-Cx43 (Sigma) antibody shows Cx43 localizing to cell-cell contacts (arrow) and in intracellular vesicles. **B.** Since the TrCx43 mutant lacks the C-terminal tail we could not use the Sigma anti-Cx43 antibody we used for detection of wild-type Cx43 or T154A. Instead we used the anti-Cx43 (Abgent) that targets the N-terminal at amino acids 107–138. We observed TrCx43 to localize to both intracellular vesicles and at cell-cell contacts, much like the control cells.

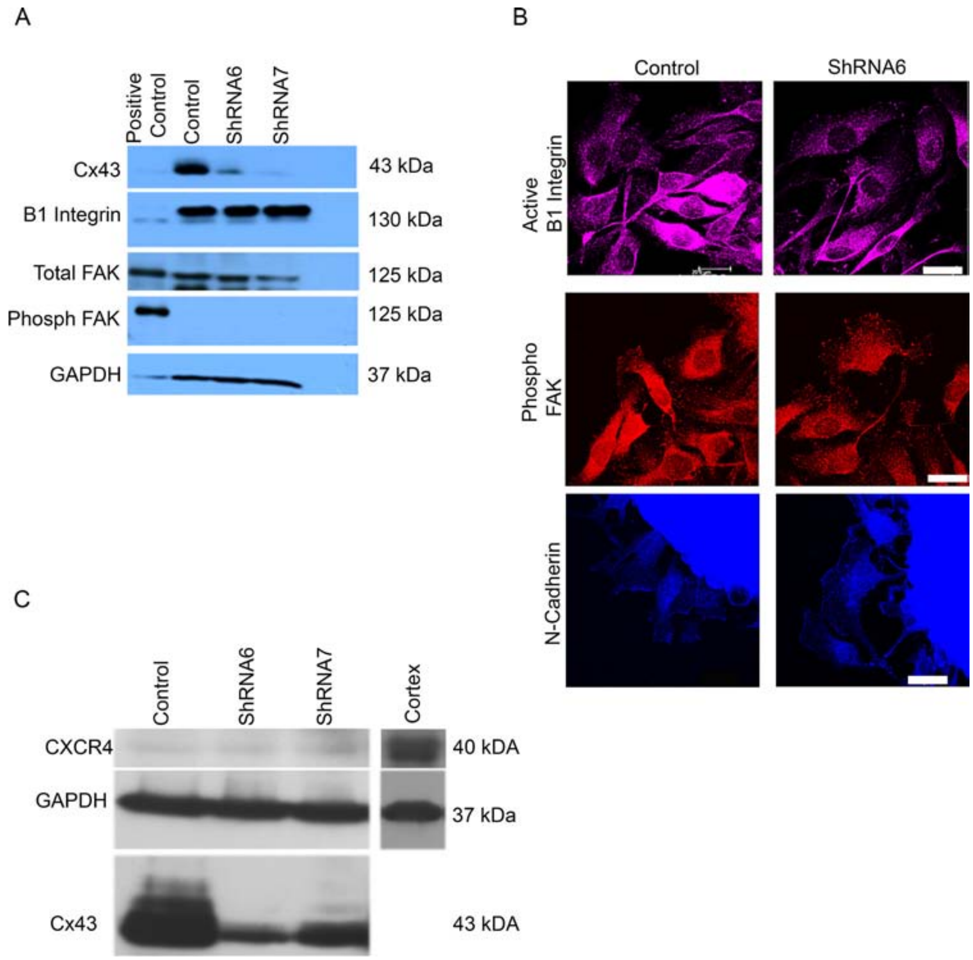

Supplementary Figure S2: Expression of cell adhesion markers are not influenced by reducing Cx43. A. Expression of cell adhesion markers, β1-Integrin (total), FAK (total), and phospho-FAK were examine by Western blot; no change in expression was observed. Ovcar3 cells were used as positive control for the adhesion markers. **B.** Immunofluorescence on control and ShRNA6 spheroids on fibronectin showed no obvious change in subcellular localization of active β1-Integrin, phospho-FAK, and N-cadherin. The thickness of the spheroid in the N-cadherin panels results in high background fluorescence. **C.** The status of CXCR4 expression does not change in ShRNA6 and ShRNA7 cells when compared to control cells. Brain cortex was used as a positive control for the antibody. These experiments were repeated twice.

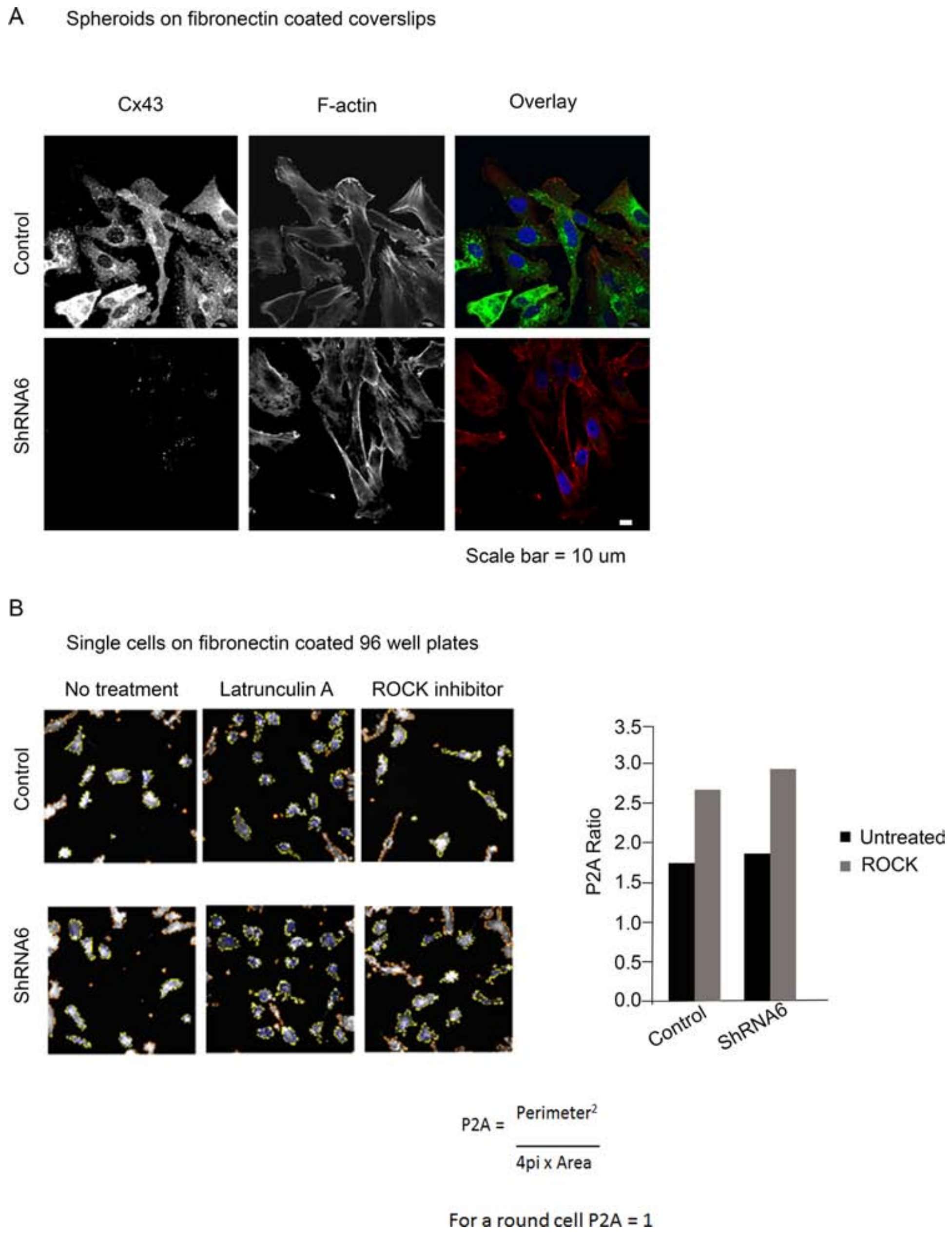

Supplementary Figure S3: Actin cytoskeleton and cell morphology are not influenced by reducing Cx43. **A.** Immunofluorescence on control and ShRNA6 spheroids on fibronectin showed no obvious change in actin cytoskeleton (phalloidin – red). **B.** Single cells were seeded at low density on fibronectin coated 96 well plates overnight. The cells were fixed and stained with phalloidin to detect actin cytoskeleton. Actin staining was used to quantify changes in cell morphology. The perimeter to area ratio (P2A) was used to quantify changes in cell shape; for a round cell P2A = 1. We did not observe changes in morphology between control and ShRNA6 cells. Rho-associated protein kinase inhibitor Y-27632 was used as a positive control to induce changes in morphology. This experiment was repeated twice.

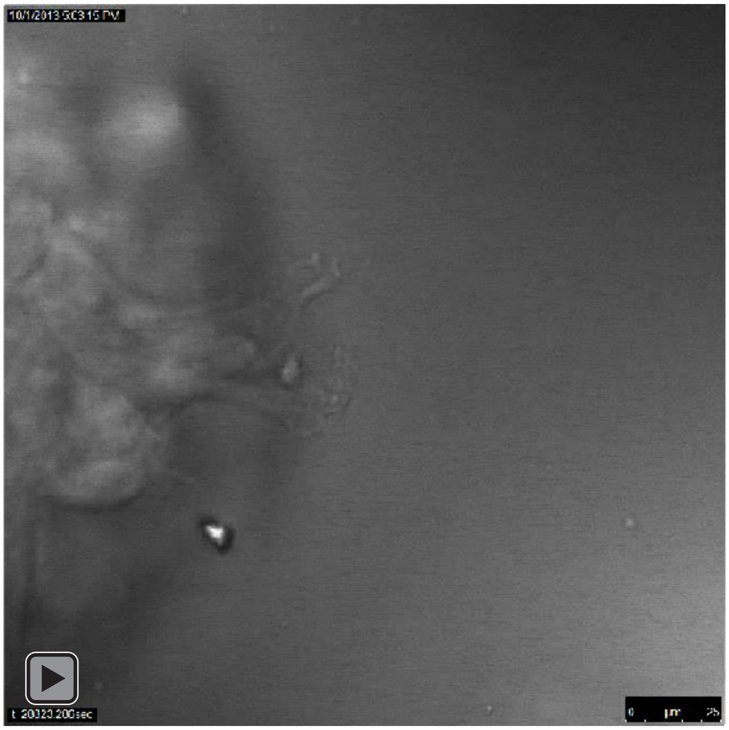

Supplementary Movie S1: Control spheroid migration assay. Control cells were cultured as spheroids for 2 days and then placed on fibronectin (10 µg/ml) coated glass bottom 35 mm dish for migration assay. After 4 hours in the incubator the spheroids were imaged at high magnification (63x objective with 1.5 zoom) for 8 hrs using a Leica SP5 confocal microscope. The spheroids were imaged in a temperature control chamber at 37°C. The spheroids were in DMEM-F12 media with B27, EGF, FGF, and HEPES. The images were acquired every 15 minutes over the 8 hrs. ImageJ plugin MTrackJ was used to draw cell tracks by following the nucleus of the cell. Cell tracks were used to quantify changes in migration patterns, speed and directionality.

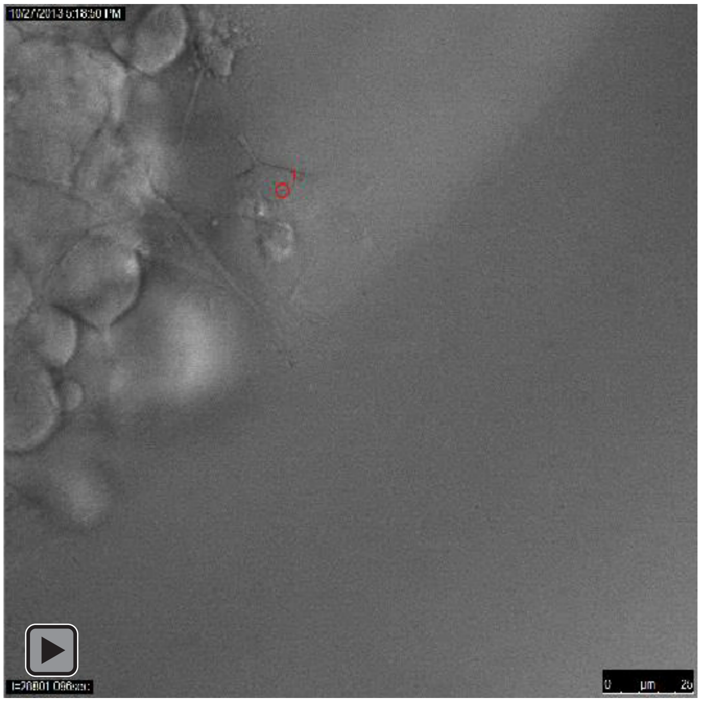

Supplementary Movie S2: ShRNA6 spheroid migration assay. ShRNA6 spheroids on fibronectin coated glass bottom 35 mm dish were imaged at high magnification for 8 hrs to monitor migration patterns. The images were acquired every 15 minutes over the 8 hrs. ImageJ plugin MTrackJ was used to draw cell tracks by following the nucleus of the cell as outlined above.
